# Therapeutic plasma exchange in the treatment of COVID-19 induced cytokine storm: the first Moroccan experience

**DOI:** 10.1186/s12879-023-08816-6

**Published:** 2023-11-25

**Authors:** Mohamed Zakaria Bouayed, Ilyass Laaribi, Iliass Benaini, Asmae Yeznasni, Sara Berrajaa, Younes Oujidi, Houssam Bkiyar, Naima Abda, Brahim Housni

**Affiliations:** 1 Anesthesia, Intensive Care and Resuscitation Department, Mohammed VI University Hospital of Oujda, Oujda, Morocco; 2 Laboratory of Epidemiology, Clinical Research and Public Health (LERCSP), Faculty of Medicine and Pharmacy, Mohammed I University, Oujda, Morocco; 3 Simulation Center, Laboratory of Anatomy, Microsurgery, Experimental Surgery and Medical Simulation (LAMESMS), Faculty of Medicine and Pharmacy, Mohammed I University, Oujda, Morocco

**Keywords:** COVID-19, Cytokine storm, Therapeutic plasma exchange, Intensive care, Mortality

## Abstract

**Introduction:**

COVID-19 induced cytokine storm is a well-documented phenomena that contributes significantly in the disease’s evolution and prognosis. Therefore, therapies such as therapeutic plasma exchange, constitute a mainstay of therapeutic management especially for critically-ill patients.

**Methods:**

We conducted a monocentric retrospective cohort study in the Resuscitation Department of the Mohammed VI University Hospital of Oujda-Morocco, to evaluate the efficiency of therapeutic plasma exchange on critically-ill COVID-19 patients over a 6 months period.

We divided our patients into two groups: patients who received TPE (Therapeutic Plasma Exchange) sessions (TPE group) and patients who only benefited from the standard protocol treatment (non TPE group).

**Results:**

Our study included a total of 165 patients, 34.5% of which benefited from TPE sessions.

We observed an improvement of oxygenation parameters (SpO2 and PaO2/FiO2 ratio) and a progressive respiratory weaning, as well as a significant decrease of biomarkers indicative of inflammation (lymphocyte count, CRP (C Reactive Protein), IL-6, Ferritin) and coagulopathy (d-dimers, fibrinogen) in the TPE group after 5 consecutive TPE sessions.

In comparison with the non-TPE group, The TPE-group patients had a shorter ICU (Intensive Care Unit) length of stay, required less frequently mechanical ventilation, and we more likely to be extubated. Furthermore, the TPE group had a lower mortality rate.

**Discussion:**

Multiple studies have reported the safety and efficiency of therapeutic plasma exchange in the COVID-19 induced cytokine storm. Given the urgent character of the pandemic at the time, each center followed its own protocol in implementing plasma exchange.

**Conclusion:**

Similar to the results reported in the literature, our study reports positive results after using TPE specifically in terms of respiratory weaning and an improvement of the cytokine storm biomarkers, and more importantly a lower mortality rate.

## Introduction

The COVID-19 health crisis has forced the world to take extreme public health measures at a level never before seen in the last century. Originating in Wuhan, China, the virus responsible for SARS-CoV-2 (Severe Acute Respiratory Syndrome Coronavirus 2) is the third highly pathogenic coronavirus after SARS-CoV-1 and MERS-CoV. To date, more than 676 million people worldwide have contracted the virus, and nearly 6.8 million deaths [1].

Given the mass of data reported in the literature, COVI D-19, initially considered a respiratory tract infection, has been redefined as a systemic disease involving multiple organs. Moreover, it has been proven that organ damage is not only due to the direct effect of the virus, but also to a disproportionate response of the immune system [2, 3], characterized by clinico-biological phenomena grouped syndromically in what is called a “cytokine storm” [4, 5].

This has opened the door to new therapeutic modalities such as immunotherapies or therapeutic plasma exchange aimed at neutralizing and/or eliminating certain agents involved in the pathological process of the cytokine storm [6, 7].

Plasma exchange therapy (TPE) has become an increasingly important part of the therapeutic armamentarium for severe forms of COVID-19, where disease progression is more likely to be attributed to the hyperinflammation and hypercoagulability tributary to the cytokine storm.

This study aims to evaluate the efficiency of therapeutic plasma exchange on critically-ill COVID-19 patients by analyzing multiple clinical and biological parameters and comparing two groups of patients.

## Methods

### Study design

This is a retrospective monocentric descriptive and analytical cohort, spread over a 6-month period, from April 2021 to September 2021.

### Study platform

This work was carried out in the Anesthesia, Intensive Care and Resuscitation Department of the Mohammed VI University Hospital of Oujda, in close collaboration with Oujda’s Faculty of Medicine Laboratory of Epidemiology, Clinical Research and Public Health (LERCSP).

Oujda’s Mohammed VI University Hospital is the largest health establishment in the Oriental region. Its Intensive Care and Resuscitation Department is composed of 5 units: polyvalent, obstetrical, pediatric, surgical and a burns resuscitation unit with a total capacity of 35 beds. During the COVID-19 pandemic, an additional capacity of 80 beds was mobilized for a total of 115 intensive care beds to manage the large flow of critical patients.

In its capacity as a tertiary care structure, the Department takes care of patients from the different hospital departments, from the ER, but also from all the hospitals of the region, as well as coordinating the inter-regional care offer. It is also the national leading department in extracorporeal circulation therapies, equipped with a total of 13 machines dedicated to Extracorporeal Membrane Oxygenation (ECMO), Renal Replacement Therapies (RRT) and Therapeutic Plasma Exchange (TPE).

### Inclusion criteria

We included:

- Confirmed COVID-19 cases
- At least 18 years of age
- With an estimated 100% CT pulmonary involvement
- With the presence of biomarkers indicative of a cytokine storm (inflammatory and hypercoagulability markers).

A confirmed case of COVID-19 was defined by a positive reverse transcription polymerase chain reaction (RT-PCR) test of a nasopharyngeal swab sample.

Biomarkers indicative of cytokine storm are:

- Inflammatory markers: hyperleukocytosis, lymphopenia, elevated C-Reactive Protein (CRP), Interleukin-6 (IL-6), and Ferritin.
- Markers of hypercoagulability: high levels of D-dimer, and Fibrinogen.

**Fig. 1:**
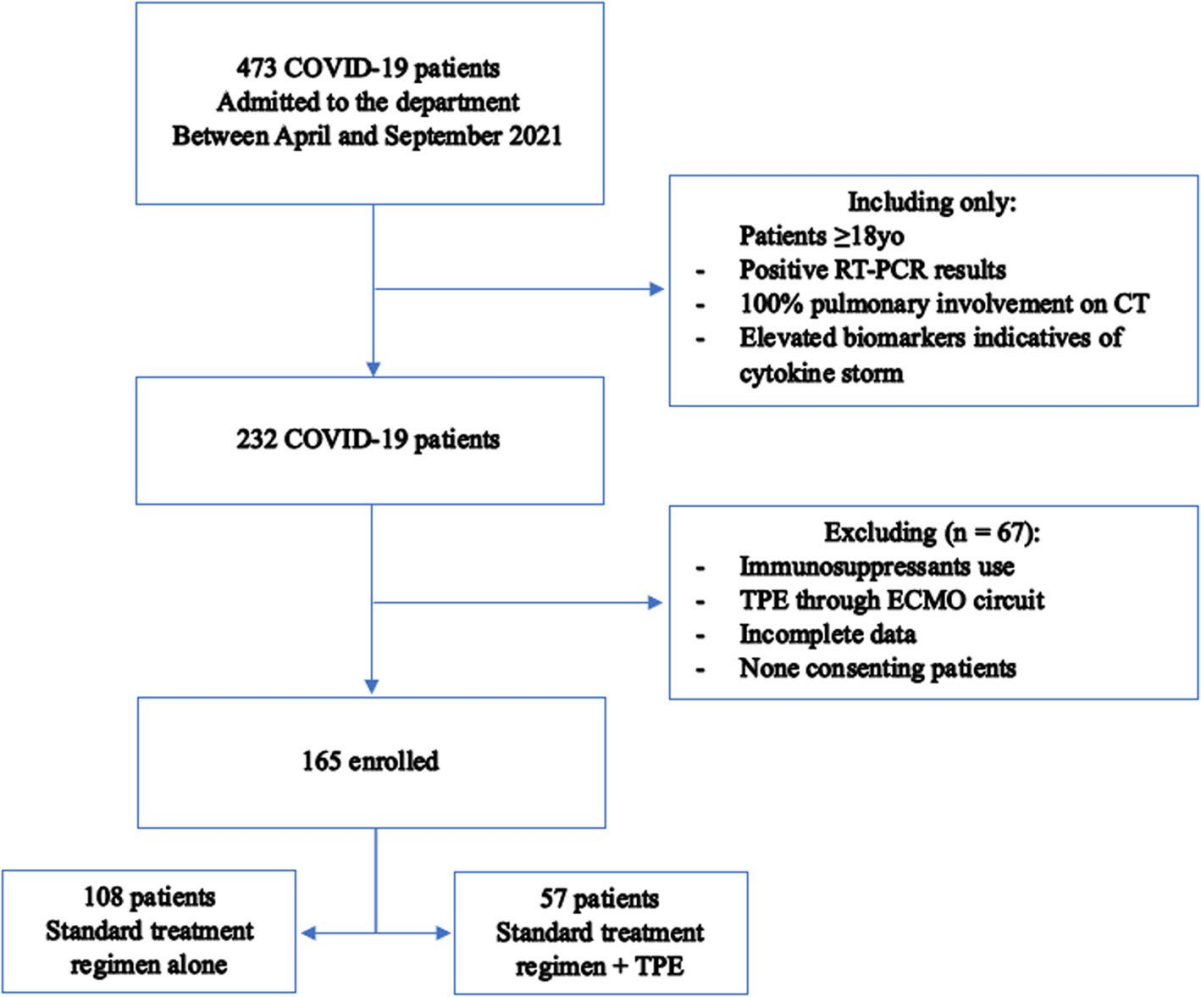
Patients selection process

The included patients were divided into two groups (Fig. 1):

- Group 1: who received the standard therapeutic protocol (vitamin therapy, corticosteroid therapy, anti-aggregation and anticoagulation, +/- antibiotic therapy) in addition to 5 TPE sessions.
- Group 2: who received only the above-mentioned standard treatment protocol, without TPE.

### Exclusion criteria

We excluded:

- Patients hospitalized for less than 48 hours (death or transfer).
- Absent or incomplete biological data.
- Patients having received one or more plasma exchange sessions in addition to immunosuppressive therapies (Tocilizumab, Infliximab).
- Patients who have received one or more plasma exchange sessions via a circuit connected to an ECMO machine (Extracorporeal Membrane Oxygenation).

### Date collection and statistical analysis

In this study, anonymized patient data were collected from their computerized records, ensuring utmost confidentiality. The collected data underwent a rigorous process, including data entry, coding, and analysis using the IBM SPSS Version 21.0 software. Initially, a comprehensive descriptive data analysis was conducted, offering detailed insights into the study sample at the point of inclusion. Categorical variables were presented as counts and percentages, while continuous variables were expressed based on their distribution: either as median values with interquartile range [IQR] for skewed data or as mean values with standard deviation (mean ± SD) for normally distributed data.

Subsequently, a meticulous analytical study was undertaken. Specifically, in Group 1, clinico-biological parameters before and after therapeutic plasma exchange (TPE) were scrutinized using a Wilcoxon test to assess significant variations. While clinico-biological parameters at the end of ICU stay and the overall evolutionary profile in both groups were comprehensively compared using a Mann-Whitney U test. Throughout the analysis, a significance level of *p* < 0.05 was adopted, denoting a stringent criterion for statistical significance.

## Results

### Descriptive study

During the specified study period, 165 patients were included in this study (Fig. 1) with a frequency of 27,5 cases/month.

The median age of our patients was 65 years old [54 – 75], ranging from 24 to 92 years old, mainly men (64,2%) with a sex ratio of 1,79.

Multiple comorbidities were reported by the patients and summarized in Table 1.

**Table 1:** Baseline characteristics

Age, median [IQR]		65 [54 – 75]
Sex (%)	Male	106 (64,2%)
	Female	59 (35,8%)
Length of stay, days (± SD)		7,83 (± 7,07)
Comorbidities, n (%)	Hypertension	55 (33,3%)
	Diabetes	50 (30,3%)
	Obesity (BMI > 30kg/m^2^)	34 (20,6%)
	Cardiopathy	22 (13,3%)
	Hypothyroidism	10 (6,1%)
	Pre-existing respiratory disease (asthma, COPD, silicosis…)	8 (4,8%)
	Cancer	5 (3,0%)
	Cerebral vascular accident	5 (3,0%)
Symptoms, n (%)	Fever	159 (96,36%)
	Dyspnea	148 (89,69%)
	Asthenia	142 (86,06%)
	Cough	121 (73,33%)
	Anosmia	114 (69,09%)
	Chills	87 (52,72%)
	Headache	58 (35,15%)
	Digestive signs	37 (22,42%)
Clinical assessment	Consciousness, n (%):	
	- GCS 15/15	148 (89,7%)
	- GCS between 9 and 14	17 (10,3%)
	- GCS ≤ 8/15	0 (0,0%)
	Blood pressure, n (%):	
	- Normal	103 (62,4%)
	- Low (BP < 90/60mmHg)	54 (32,7%)
	- Shock (BP < 96/60mmHg + Lactate > 2mmol/L)	8 (2,9%)
	Respiratory parameters	
	- SpO_2_ (%), mean ± SD	82 ± 7
	- PaO_2_/FiO_2_ ratio (mmHg), median [IQR]	145 [127 – 166]
Biological findings	WBC (× 10^3^/μL), mean ± SD	11,40 ± 6,07
	High WBC, (%)	50,9%
	Lymphocytes (× 10^3^/μL), median [IQR]	0,75 [0,52 – 1,09]
	Lymphopenia (%)	88,48%
	CRP (mg/L), median [IQR]	176 [115 – 255]
	High CRP level (%)	100%
	IL-6 (pg/mL), median [IQR]	257 [110 – 692]
	High IL-6 level (%)	90,9%
	Ferritin (ng/mL), median [IQR]	1227 [468 – 2209]
	Hyperferritinemia (%)	91,5%
	D-dimers (ng/mL), median [IQR]	755 [565 – 959]
	High D-dimers level (%)	98,7%
	Fibrinogen (g/L), median [IQR]	6,6 [4, 9 – 7, 5]
	High fibrinogen level (%)	87,8%
Radiological findings	Degree of pulmonary involvement on CT scan (%)	100%
	Pulmonary embolism, n (%)	29 (17,57%)

Upon admission, an assessment of the respiratory status judged based on the SpO_2_ and PaO_2_/FiO_2_ ratio, hemodynamic status based on arterial pressure, and neurological status based on the Glasgow Coma Scale, were carried out, summarized in Table 1.

Laboratory data: initial blood work revealed a high WBC (White Blood Cells) in 50,9%, lymphopenia in 88,48%, high CRP and IL-6 levels in 100% and 90,9% respectively, hyperferritinemia in 91,5%, as well as high d-dimers and fibrinogen levels in 98,7% and 87,8% respectively.

As an inclusion criterion, all cases had an estimated degree of pulmonary involvement of 100%.

A contrast chest CT was performed in 47 patients (28.48% of cases). A pulmonary embolism was found in 29 patients (17.57%).

Biological and radiological data are compiled in Table 1.

Treatments and Outcome: all patients required oxygen and/or ventilation support adapted according to their needs and response varying from nasal oxygen therapy to mechanical ventilation. Throughout hospitalization, 95 patients required mechanical ventilation, out of whom only 10 were extubated.

All patients underwent a similar medication regimen including curative anticoagulation using sodic enoxaparin doses adapted to renal function, antiplatelet aggregation therapy using acetylsalicylic acid 160mg/day, corticosteroids using dexamethasone 6mg/day, vitamin C 2000mg/day, vitamin D 25000UI/week, zinc 45mg/day, and proton pomp inhibitor 40mg/day (Table 2).

**Table 2:** Treatments and outcome

Treatments	Oxygen and ventilation support, n (%)	
	- Nasal oxygen therapy	23 (13,9%)
	- High concentration oxygen therapy	67 (42,4%)
	- High flow nasal oxygen therapy	99 (60,0%)
	- Noninvasive ventilation	45 (27,2%)
	- Mechanical ventilation	95 (57,6%)
	Corticosteroids, n (%)	
	Dexamethasone 6mg/d	165 (100%)
	Anticoagulation, n (%)	
	- Enoxaparine	165 (100%)
	Anti-platelet aggregation therapy, n (%)	
	- Acetylsalicylic acid	165 (100%)
	Adjuvant therapies:	
	Vitamin C 2000mg/d	165 (100%)
	Vitamin D 25000UI/week	165 (100%)
	Zinc 45mg/d	165 (100%)
	Proton pomp inhibitor 40mg/d	165 (100%)
	Therapeutic plasma exchange, n (%)	57 (34,5%)
	Vascular access:	
	- Jugular	19 (33,3%)
	- Femoral	38 (66,7%)
	Substitution fluid:	
	- Fresh Frozen Plasma	100%
	Exchange volume (mL), mean ± SD	2915 ± 338
	Circuit anticoagulation:	
	- Non-Fractioned Heparin	100%
	Number of sessions, n	5
	Session length of time (min), mean ± SD	119 ± 24
Outcome	Length of stay (days), median [IQR]	15 [11 – 24]
	Extubation rate, n (%)	10 (10,5%)
	Survival rate, n (%)	80 (48,5%)
	Mortality rate, n (%)	85 (51,5%)

Depending on patient’s approval and technical availability, 57 patients benefited from a total of five therapeutic plasma exchange sessions, through a central jugular or femoral venous line, using FFP (Fresh Frozen Plasma) as substitution fluid with a mean volume exchange of 2915 ± 338mL, and NFH (Non-Fractioned Heparin) for circuit anticoagulation.

The median length of stay was 15 days [11 – 24], with a survival rate of 48,5%.

Treatments and Outcome are summarized in Table 2.

### Analytical study

We first compared the clinical and biological data of the TPE group before (admission) and after (end of ICU stay) 5 consecutive of therapeutic plasma exchange sessions (Table 3).

**Table 3:** Evolution of clinical and biological parameters before and after TPE

Variables	Pre-TPE	Post-TPE	*p* value
SpO_2_ (%)	80 ± 7	90 ± 4	** < 0,0001**
PaO_2_/FiO_2_ (mmHg)	142 [125 – 159]	202 [180 – 235]	** < 0,0001**
WBC (10^3^/μL)	10,00 ± 54,66	10,74 ± 64,25	0,354
Lymphocytes (× 10^3^/μL)	0,67 [0,52 – 1,00]	0,97 [0,64 – 1,32]	** < 0,0001**
CRP (mg/L)	176 [131 – 286]	108 [72 – 175]	** < 0,0001**
IL-6 (pg/mL)	423 [166 – 1750]	57 [20 – 148]	** < 0,0001**
Ferritin (ng/mL)	1804 [611 – 3041]	1068 [305 – 2260]	** < 0,0001**
D-dimers (ng/mL)	726 [533 – 946]	402 [178 – 767]	** < 0,0001**
Fibrinogen (g/L)	6,2 [4,3 – 7,7]	4,9 [3,3 – 6,3]	** < 0,0001**

We observed a statistically significant improvement in SpO_2_ and PaO_2_/FiO_2_ ratio (Table 3).

We also observed a significant decrease in inflammatory and hypercoagulability biomarkers indicative of a cytokine storm, including significantly reduced levels of CRP, IL-6, ferritin, D-dimer and fibrinogenemia, as well as an increase in lymphocyte count. However, there was no statistically significant difference in leukocyte count before and after plasma exchange (Table 3).

**Fig. 2:**
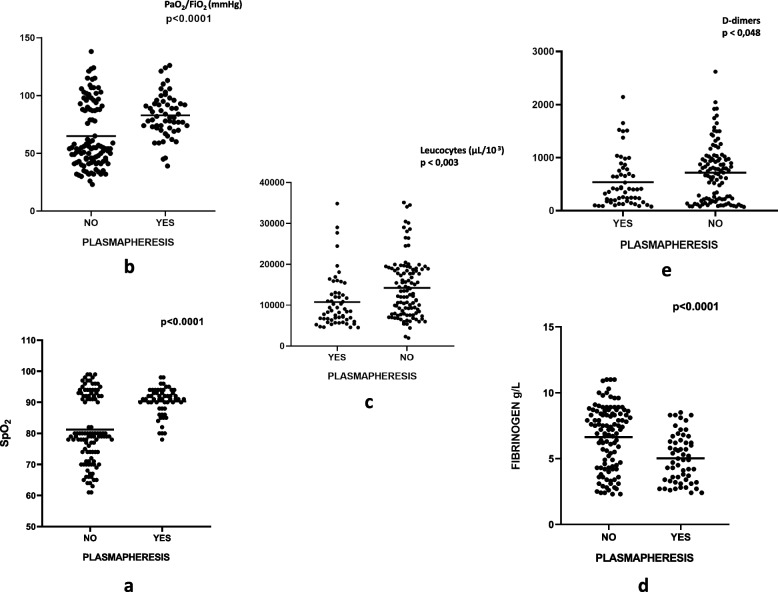
Distribution of clinically and biologically significant parameters following treatment in the two groups: SpO_2_ (**a**), PaO_2_/FiO_2_ (**b**), WBC (**c**), Fibrinogen (**d**), and d-dimers (**e**)

Our analysis also focused on an event-based comparison of the clinical and biological parameters of the two groups, the event being the end of ICU stay (Discharge, Transfer to a none-ICU unit, Death). We observed a significant improvement in respiratory parameters in group 1 compared to group 2, namely, an average SpO2 of 90 ± 4% vs 81 ± 11% (*p* < 0.0001) and a median PaO2/FiO2 ratio of 202mmHg [180 – 235] vs 175mmHg [123 – 231] (*p* < 0.0001). We also noted a progressive respiratory weaning from high-flow oxygen therapy and non-invasive ventilation in 61.4% of cases in group 1 compared to 32.4% in group 2. Conversely, 67.6% of patients in group 2 required mechanical ventilation compared to only 38.6% of patients in group 1 (*p* < 0.0001). In terms of biology, leukocyte, D-dimer, and fibrinogen levels were significantly lower in group 1 compared to group 2 (Table 4, Fig. 2).

**Table 4:** Comparison of clinical and biological parameters between both groups

Variables	**Group 1**	**Group 2**	*p* value
SpO_2_ (%)	90 ± 4	81 ± 11	** < 0,0001**
PaO_2_/FiO_2_ (mmHg)	202 [180 – 235]	175 [123 – 231]	** < 0,0001**
WBC (10^3^/μL)	10,74 ± 6,42	14,22 ± 7,26	**0,003**
Lymphocytes (× 10^3^/μL)	0,97 [0,64 – 1,32]	0,84 [0,46 – 1,35]	0,175
CRP (mg/L)	108 [72 – 175]	176 [115 – 255]	0,07
IL-6 (pg/mL)	57 [20 – 148]	126 [18 – 535]	0,365
Ferritin (ng/mL)	1068 [305 – 2260]	1179 [302 – 2306]	0,939
D-dimers (ng/mL)	402 [178 – 767]	629 [198 – 959]	**0,048**
Fibrinogen (g/L)	4,9 [3,3 – 6,3]	6,2 [4,0 – 8,1]	** < 0,0001**

The patients in group 1 had a shorter stay in the intensive care unit (ICU) compared to those in group 2, with a median length of stay of 13 days [8–19] versus 16 days [12–27] (*p* = 0,006), respectively. Also, only 22 patients (38.6%) in group 1 required mechanical ventilation, compared to 73 patients (67.6%) in second group (*p* < 0,0001). Additionally, patients in group 1 were more likely to be extubated than those in group 2, with 7 patients (31.8%) compared to 3 patients (4.1%), respectively (*p* = 0,002). Furthermore, we report a lower mortality rate in group 1 (26,3%) compared to group 2 (64,8%) (*p* < 0,0001).

## Discussion

In our study, a statistically significant improvement of clinical (SpO_2_ and PaO_2_/FiO_2_ ratio) and biological (Lymphocytes, CRP, IL-6, Ferritin, D-dimers, Fibrinogen) parameters was attributed to the use of therapeutic plasma exchange in the first group. Additionally, a statistically significant difference was observed comparing clinical (SpO_2_ and PaO_2_/FiO_2_ ratio) and biological (WBC, D-dimers, Fibrinogen) between both groups included in this study, as well as a higher extubation rate, a lower ICU length of stay and mortality in group 1.

Beyond its pulmonary tropism, COVID-19 is a systemic disease with multi-organ involvement attributed to an excessive immune response to the virus [20, 21]. In fact, this hyperinflammatory induced state has been well documented and corresponds to the "cytokine storm" syndrome previously described in various conditions [8, 9].

Serum cytokine levels that are elevated in patients with COVID-19-associated cytokine storm include interleukin-1β, interleukin-6, IP-10 (Interferon gamma-induced protein 10), TNF (Tumor Necrosis Factor), interferon-γ, MIP-1α and 1β (Macrophage Inflammatory Protein-1 Alpha and 1 Beta) proteins, and VEGF (Vascular Endothelial Growth Factor) [10, 21]. Higher levels of interleukin-6 are strongly associated with shorter survival [11]. Circulating activated CD4 + and CD8 + (Cluster Of Differentiation 4 and 8) T cell and plasmablast levels are also increased in COVID-19 [12].

In addition to elevated systemic cytokine levels and activated immune cells, several clinical and laboratory abnormalities, such as elevated CRP and d-dimer levels, hypoalbuminemia, renal dysfunction, and effusions, are also observed in COVID-19. These organ failures and biological abnormalities reflect the degree of hyperinflammation and tissue damage and can predict the prognosis of COVID-19 [13].

Pre-existing comorbidities such as hypertension, diabetes, and obesity are associated with more severe forms of COVID-19 [14], perhaps due to the pre-existing chronic inflammatory state or a lower threshold for the development of organ dysfunction due to the immune response [5].

Naturally, therapies targeting hyperinflammation, such as immunosuppressants [15], and therapeutic plasma exchange therapy (given their proven role in blood purification by eliminating high molecular weight circulating substances and restoring homeostasis in many dysregulated biological pathways [16]), have a valuable place in the therapeutic arsenal against COVID-19.

In a multicenter case-control study was conducted by Gucyetmez et al. [17] to determine the effectiveness of TPE in patients with COVID-19 admitted to five ICUs in Turkey. The patients were divided into two groups: group 1 consisted of 18 patients who received three consecutive TPE sessions, and group 2 consisted of 35 patients who only received standard therapeutic protocol. The mean SpO2 in group 1 was 91 ± 7% vs. 89 ± 5% in group 2. The same study also reported a mean pre-TPE WBC count of 9.08 ± 4.1 × 103/μL compared to a mean post-TPE WBC count of 9.14 ± 3.5 × 103/μL. To date, the study by Gucyetmez et al. and ours are the only ones to have included SpO2 and WBC count as variables to study the effectiveness TPE in COVID-19.

Similarly, to our findings, a randomized control trial including 87 patients divided into two groups [18], depending on who benefited from TPE on top of the standard therapeutic protocol or just the latter, and in which significant improvement in PaO_2_/FiO_2_ ratio, CRP, IL-6, Ferritine, and D-dimers were noted before and after TPE sessions. The same study reported better clinical and biological parameters in the intervention group comparted to the control group.

While the efficiency of TPE has extensively documented, the diversity of methods used by each center has been well documented, particularly in the literature review conducted by Krzych et al. [6] and later by Beraud et al. [19], the latter of which included 34 articles (1 randomized controlled trial, 4 case–control studies, 15 case series, and 14 case reports, totaling 267 patients treated with plasma TPE).

We highlighted three major differences. First, the number of sessions varied from 1 to 9 sessions depending on the case series in question. Second, there was a variability in the choice of replacement fluid, with Fresh Frozen Plasma (FFP) being the most commonly used, followed by 5% albumin. Finally, there was a predominance of regional citrate anticoagulation (RCA) [6, 19].

In the absence of guidelines regarding the practical aspects of TPE and following the department’s procedural habits, we opted for a number of 5 consecutives TPE sessions using FFP as a substitution fluid and heparin for anticoagulation given that RCA is not available. The therapy was provided to patients based on the informed consent of either the patient or a proxy, and also based on the therapy’s availability given the limited number of machines, consumables, and FFP.

Taking into account the therapy’s procedural diversity, a multinational team of the International Society of Blood Transfusion (ISBT) conducted a literature review relying on the recommendations of the American Society for Apheresis (ASFA) to formulate preliminary clinical practice recommendations related to the performance of plasma exchanges in COVID-19 [22], which concluded up to the date of its publication that the use of TPE in COVID-19-induced cytokine storm is categorized as Class III, Grade 2B, meaning that its optimal role is not established, and that the quality of evidence evaluated at that time supported only a weak overall recommendation for this approach, indicated in critically-ill COVID-19 patients with virtually no absolute contraindications, initiated early in the disease progression, using FFP or ideally convalescent plasma for substitution, and RCA for anticoagulation. The recommended exchange volume is 1 to 1.5 times the patient’s TPV (Total Plasma Volume), for virtually as many sessions as necessary.

The results of our cohort are in line with those widely reported by several studies already in the literature, advocating for the effectiveness of plasma exchange in COVID-19, especially in severely ill patients requiring ICU care.

The COVID-19 pandemic proved to be a unique experience, opening the door to an unlimited potential of research and experimentation. While our study could have been better led, with more clinical and biological parameters monitored, it offers ad significant sample size, with valuable results.

That said, further studies are needed to first describe more specifically and closely delineate the clinical-biological spectrum of the cytokine storm induced by COVID-19, particularly its often-neglected extrapulmonary manifestations, but also to support the safety and efficacy of plasma exchange in COVID-19.

In this sense, it would be preferable to evaluate the use of plasma exchange alone, or in combination with other therapies, for COVID-19 patients in the context of prospective, randomized, and controlled clinical trials. This approach could yield fruitful results in saving lives and paving the way for future consideration of plasma exchange in similar diseases.

Our study has a number of strengths including a specific focus on the use of TPE in critically-ill patients, joining only a limited number of studies published to this day, as well as comparative approach comparing outcomes before and after TPE sessions within Group 1 and conducting an event-based comparison between both groups, enhancing the depth of the analysis. This Event-Based Comparison provides a meaningful endpoint, aligning with practical clinical outcomes. This study also carries certain weaknesses such as its retrospective and single-center design. Also, a longitudinal data analysis comparing parameters and outcomes at various time points, accounting for individual variations would have been more informative, specifically on the efficiency of TPE with less than 5 sessions.

## Conclusion

The change that occurred in the definition of COVID-19 from a viral pneumonia to a multiphasic systemic disease with multiple organ involvement has significantly impacted the clinical and therapeutic approach to the disease. COVID-19 has highlighted the essential role of an effective host immune response and the devastating effect of immune dysregulation.

Several promising therapies have found their place in COVID-19, but there is currently no definitive cure. Based on the general practice of apheresis therapies and the pathophysiology of the cytokine storm induced by COVID-19, it is reasonable to consider therapeutic plasma exchange as a potential therapeutic modality in this context, although it would be more prudent to consider this therapy on an individual basis.

## Data Availability

Anonymized data may be made available upon request. Author to contact: Dr. Mohamed Zakaria BOUAYED.
