# Correction: Therapeutic plasma exchange in the treatment of COVID-19 induced cytokine storm: the first Moroccan experience

**DOI:** 10.1186/s12879-023-08855-z

**Published:** 2023-12-08

**Authors:** Mohamed Zakaria Bouayed, Ilyass Laaribi, Iliass Benaini, Asmae Yeznasni, Sara Berrajaa, Younes Oujidi, Houssam Bkiyar, Naima Abda, Brahim Housni

**Affiliations:** 1 Anesthesia, Intensive Care and Resuscitation Department, Mohammed VI University Hospital of Oujda, Oujda, Morocco; 2 Laboratory of Epidemiology, Clinical Research and Public Health (LERCSP), Faculty of Medicine and Pharmacy, Mohammed I University, Oujda, Morocco; 3 Simulation Center, Laboratory of Anatomy, Microsurgery, Experimental Surgery and Medical Simulation (LAMESMS), Faculty of Medicine and Pharmacy, Mohammed I University, Oujda, Morocco

**BMC Infectious Diseases (2023) 23:829**

10.1186/s12879-023-08816-6

The original publication of this article contained an incorrect author name. The incorrect and correct information is shown in this correction article. The affiliation of Naima Abda has also been amended.

Incorrect

Asmae Yeznasn

Correct

Asmae Yeznasni

The original article has been updated.

